# Supplementary material for: Volumetric Absorptive Microsampling of Saliva for Pharmacokinetic Evaluation of Mycophenolic Acid and Its Glucuronide Metabolite in Pediatric Renal Transplant Recipients: Bioanalytical Method Validation and Clinical Feasibility Evaluation
Source: Pharmaceuticals (Basel). 2025 Nov 17;18(11):1744. doi: 10.3390/ph18111744 (PMC12655819; doi:10.3390/ph18111744)
Supplement: Supplementary file 1 [file pharmaceuticals-18-01744-s001.zip › pharmaceuticals-3953387-supplementary.pdf]

# SUPPLEMENTARY FILE

**VOLUMETRIC ABSORPTIVE MICROSAMPLING OF SALIVA FOR PHARMACOKINETIC EVALUATION OF MYCOPHENOLIC ACID AND ITS GLUCURONIDE METABOLITE IN PEDIATRIC RENAL TRANSPLANT RECIPIENTS: BIOANALYTICAL METHOD VALIDATION AND CLINICAL FEASIBILITY EVALUATION**

Arkadiusz Kocur[1*], Joanna Sobiak[2], Agnieszka Czajkowska[3], Jacek Rubik[4] and Tomasz Pawiński[1]

[1] Department of Drug Chemistry, Pharmaceutical and Biomedical Analysis, Medical University of Warsaw, Banacha 1, 02-097 Warsaw, Poland.

[2] Department of Physical Pharmacy and Pharmacokinetics, Poznan University of Medical Sciences, Rokietnicka 3, 60-806 Poznan, Poland.

[3] Therapeutic Drug Monitoring, Clinical Pharmacokinetics and Toxicology Laboratory, Department of Clinical Biochemistry, The Children's Memorial Health Institute, Dzieci Polskich 20, 04-730 Warsaw, Poland.

[4] Department of Nephrology, Kidney Transplantation and Arterial Hypertension, The Children's Memorial Health Institute, Dzieci Polskich 20, 04-730 Warsaw, Poland.

Corresponding author: Arkadiusz Kocur, arkadiusz.kocur@wum.edu.pl

**ORCID identifiers:**

- 0000-0002-4833-8532 (AK)
- 0000-0002-2764-1575 (JS)
- 0000-0003-0199-5751 (AC)
- 0000-0002-3392-2154 (JR)
- 0000-0001-9110-4312 (TP)

**Table S1.** Results of matrix effect (ME), absolute recovery (AR), and process efficiency (PE) evaluation for the normalized analyte to IS ratio [n=6].

| LEVEL | ME [%][a] | | AR [%][a] | | PE [%][a] | |
|---|---|---|---|---|---|---|
| | MPA/IS ratio[b] | MPAG/IS ratio[b] | MPA | MPAG | MPA | MPAG |
| **LQC** | 1.10 (5.41) | 1.05 (7.27) | 71.44 (4.83) | 79.98 (6.62) | 69.21 (5.87) | 57.33 (6.25) |
| **HQC** | 0.92 (3.13) | 0.90 (4.06) | 82.56 (2.41) | 84.21 (3.01) | 75.23 (4.10) | 60.89 (5.28) |

[a] Data expressed as mean value (with CV%). [b] Normalized matrix factor (MPAG or MPAG-to-IS matrix factor ratio).

**Table S2.** Results of stability evaluation under various conditions for Mitra™ VAMS saliva samples expressed as mean recoveries [n=6].

| LEVEL | vsMPA | | | | vsMPAG | | | |
|---|---|---|---|---|---|---|---|---|
| | Room temperature (light-protected with desiccant) | | | | Room temperature (with daily light exposure) | | | |
| | 24 hours | 3 days | 1 week | 2 weeks | 24 hours | 3 days | 1 week | 2 weeks |
| **LQC** | 101.13% | 99.14% | 98.01% | 95.57% | 103.15% | 100.46% | 99.67% | 97.25% |
| **HQC** | 99.89% | 100.24% | 97.15% | 95.98% | 100.16% | 98.94% | 96.66% | 94.51% |

LQC – low quality control; HQC – high quality control; vsMPA – mycophenolic acid level in dried saliva sample collected using volumetric absorptive microsampling; vsMPAG – mycophenolic acid glucuronide level in dried saliva sample collected using volumetric absorptive microsampling.

**Table S3.** Summarized results of the MPA and MPAG quantification in each type of matrix in the study group (n=20).

| | $C_0$ | $C_{0.5}$ | $C_1$ | $C_2$ | $C_4$ |
|---|---|---|---|---|---|
| fMPA [mg/L] | 0.022±0.016 (0.003-0.071) | 0.104±0.125 (0.009-0.531) | 0.149±0.146 (0.023-0.566) | 0.049±0.041 (0.005-0.133) | 0.031±0.050 (0.001-0.230) |
| tMPA [mg/L] | 2.151±1.620 (0.352-7.976) | 10.722±9.559 (1.172-36.646) | 11.377±6.809 (3.026-26.332) | 4.685±3.412 (0.788-13.095) | 2.226±2.389 (0.672-11.499) |
| sMPA [µg/L] | 8.242±5.662 (2.532-23.543) | 27.151±7.858 (6.184-82.349) | 51.604±45.799 (7.956-219.042) | 17.737±16.069 (5.462-73.803) | 8.004±9.492 (1.046-32.396) |
| vsMPA [µg/L] | 7.535±4.890 (2.133-20.877) | 27.720±9.143 (5.442-89.244) | 52.246±40.870 (8.097-245.77) | 17.136±16.660 (5.330-76.264) | 7.252±8.031 (1.21-28.993) |
| vMPA [mg/L] | 1.803±1.298 (0.588-6.640) | 9.660±8.961 (1.788-34.021) | 10.087±5.973 (3.118-26.805) | 4.430±2.995 (0.660-12.810) | 1.772±2.007 (0.467-9.696) |
| fMPAG [mg/L] | 3.893±2.073 (1.411-9.219) | 6.264±4.238 (2.611-19.809) | 8.029±4.393 (2.432-18.563) | 6.889±3.791 (3.232-15.633) | 4.701±2.218 (1.316-10.020) |
| tMPAG [mg/L] | 23.805±12.32 (8.799-51.217) | 37.800±17.081 (14.792-79.638) | 48.647±22.134 (19.238-109.280) | 34.258±13.977 (15.527-78.165) | 27.075±12.413 (8.400-58.891) |
| sMPAG [µg/L] | 19.135±12.712 (5.788-54.772) | 39.659±26.611 (5.937-116.48) | 70.173±44.943 (13.463-146.000) | 39.371±25.210 (13.424-104.100) | 22.417±15.097 (6.887-55.787) |
| vsMPAG [µg/L] | 17.834±12.122 (5.665-49.255) | 37.704±24.598 (6.099-110.64) | 67.422±44.462 (15.433-146.54) | 39.258±25.317 (10.665-88.543) | 19.762±13.638 (5.322-53.660) |
| vMPAG [mg/L] | 22.481±10.350 (11.370-47.394) | 34.340±14.354 (14.993-69.373) | 44.003±20.520 (19.796-110.756) | 34.039±12.356 (15.977-56.664) | 25.381±11.908 (8.468-48.160) |

Values are expressed as mean with SD and range. fMPA– free mycophenolic acid level; fMPAG- free mycophenolic acid glucuronide level; tMPA – total mycophenolic acid level; tMPAG – total mycophenolic acid glucuronide level; sMPA – wet salivary mycophenolic acid level; sMPAG – wet salivary mycophenolic acid glucuronide level; vsMPA – 'dried' salivary mycophenolic acid level; vsMPAG – 'dried' salivary mycophenolic acid glucuronide level; vMPA– capillary blood (VAMS) mycophenolic acid level; vMPAG– capillary blood (VAMS) mycophenolic acid glucuronide level.

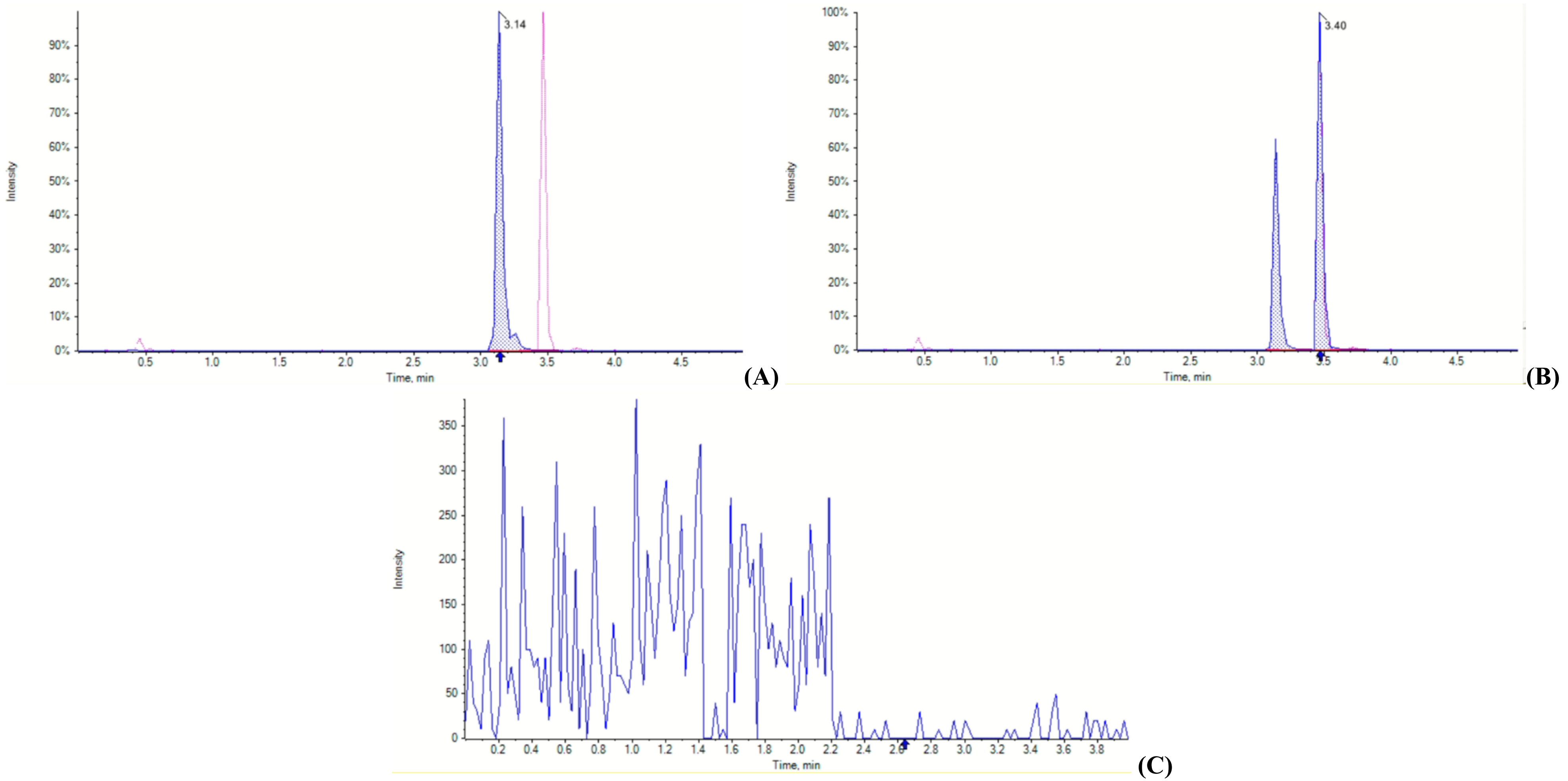


**Figure S1.** Representative chromatograms for: (A) LLOQ of MPAG in dried saliva sample (pink peak: SIL-IS, blue peak: MPAG); (B) LLOQ of MPA in dried saliva (first peak is MPA from MPAG dissociation in ion source, the second is the exact peak of MPA); (C) blank dried saliva sample chromatogram.

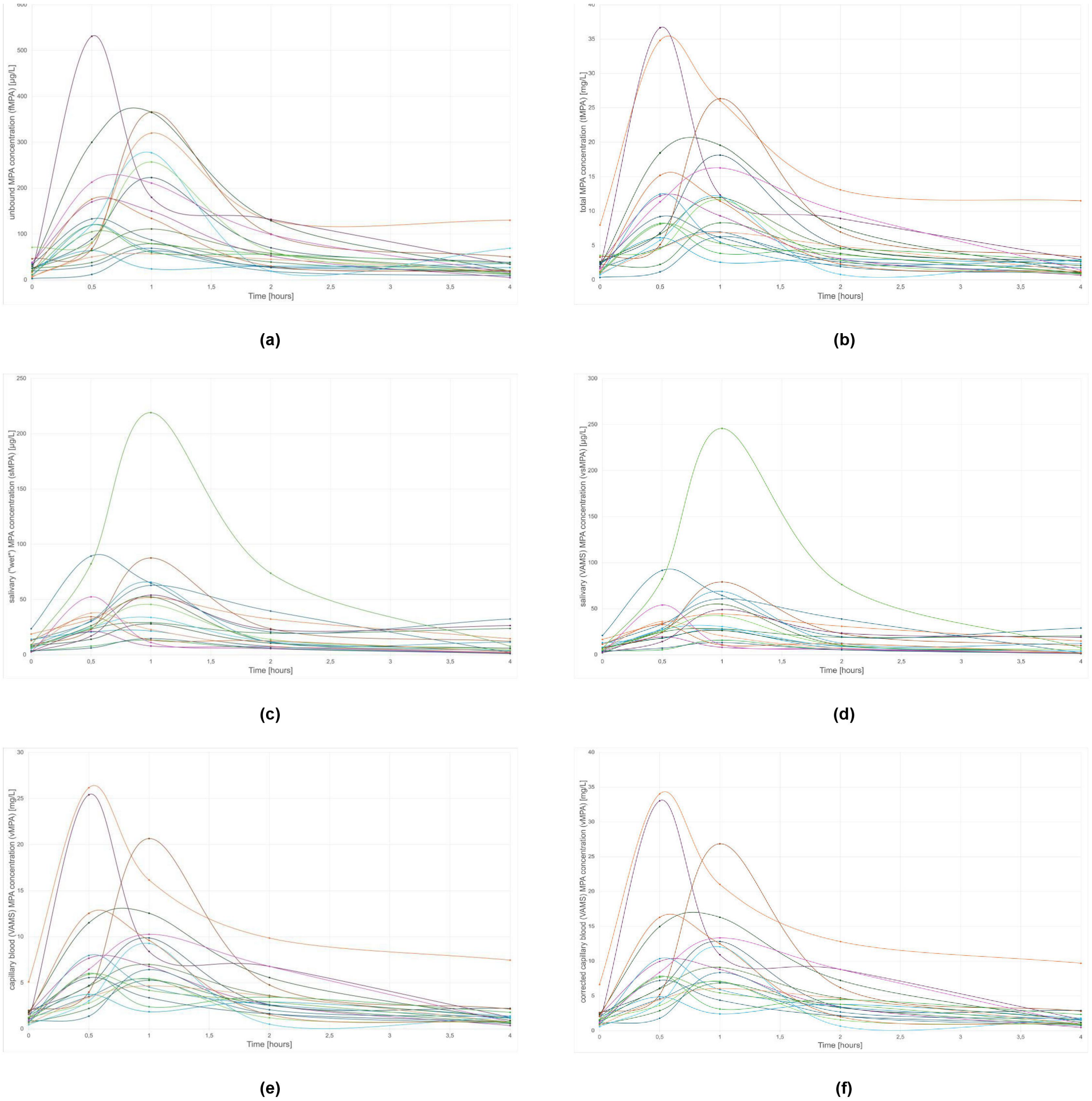


**Figure S2.** Visualization of pharmacokinetics profiles for MPA of all patients included in the study: (a) free MPA levels, fMPA; (b) total MPA levels, tMPA; (c) wet salivary MPA levels, sMPA; (d) dried salivary MPA levels, vsMPA; (e) capillary blood MPA levels; (f) corrected capillary blood MPA levels, vMPA.

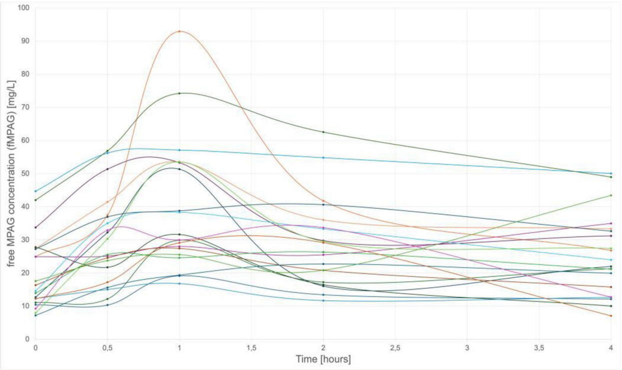


**(a)**

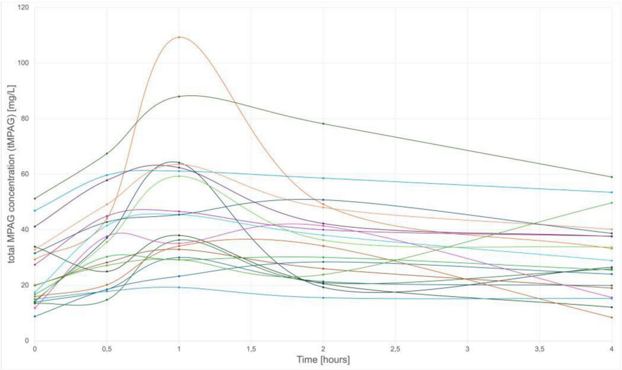


**(b)**

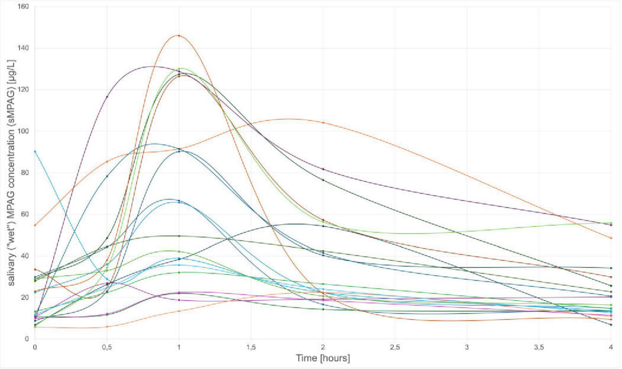


**(c)**

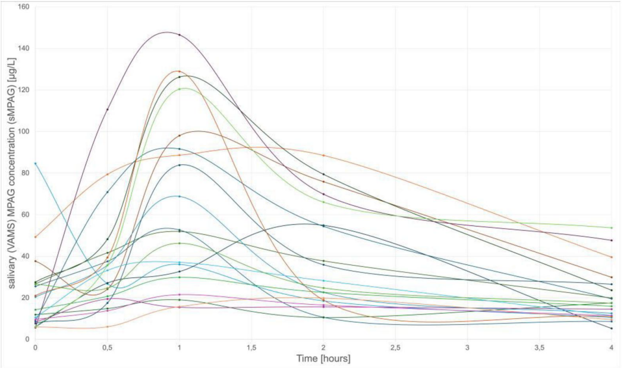


**(d)**

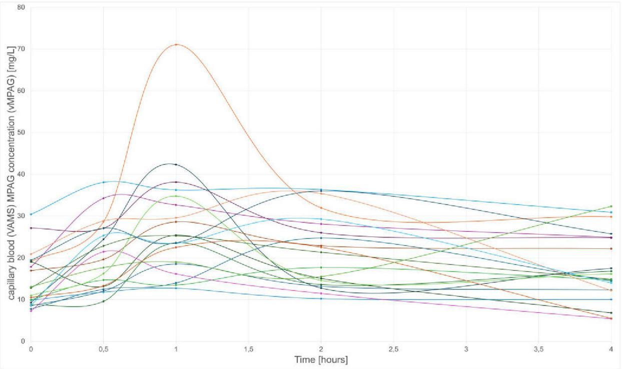


**(e)**

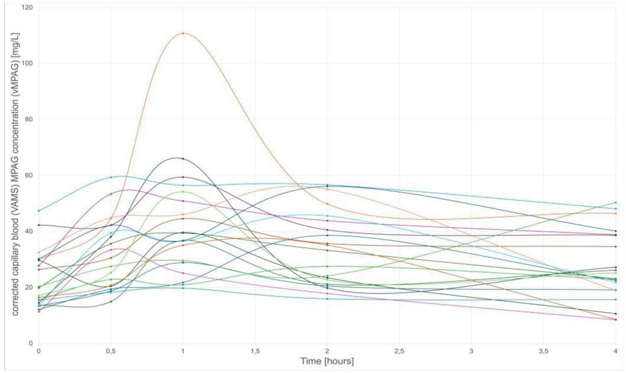


**(f)**

**Figure S3.** Visualization of pharmacokinetics profiles for MPAG of all patients included in the study: (a) free MPAG levels, fMPAG; (b) total MPAG levels, tMPAG; (c) wet salivary MPAG levels, sMPAG; (d) dried salivary MPAG levels, vsMPAG; (e) capillary blood MPAG levels; (f) corrected capillary blood MPAG levels, vMPAG.

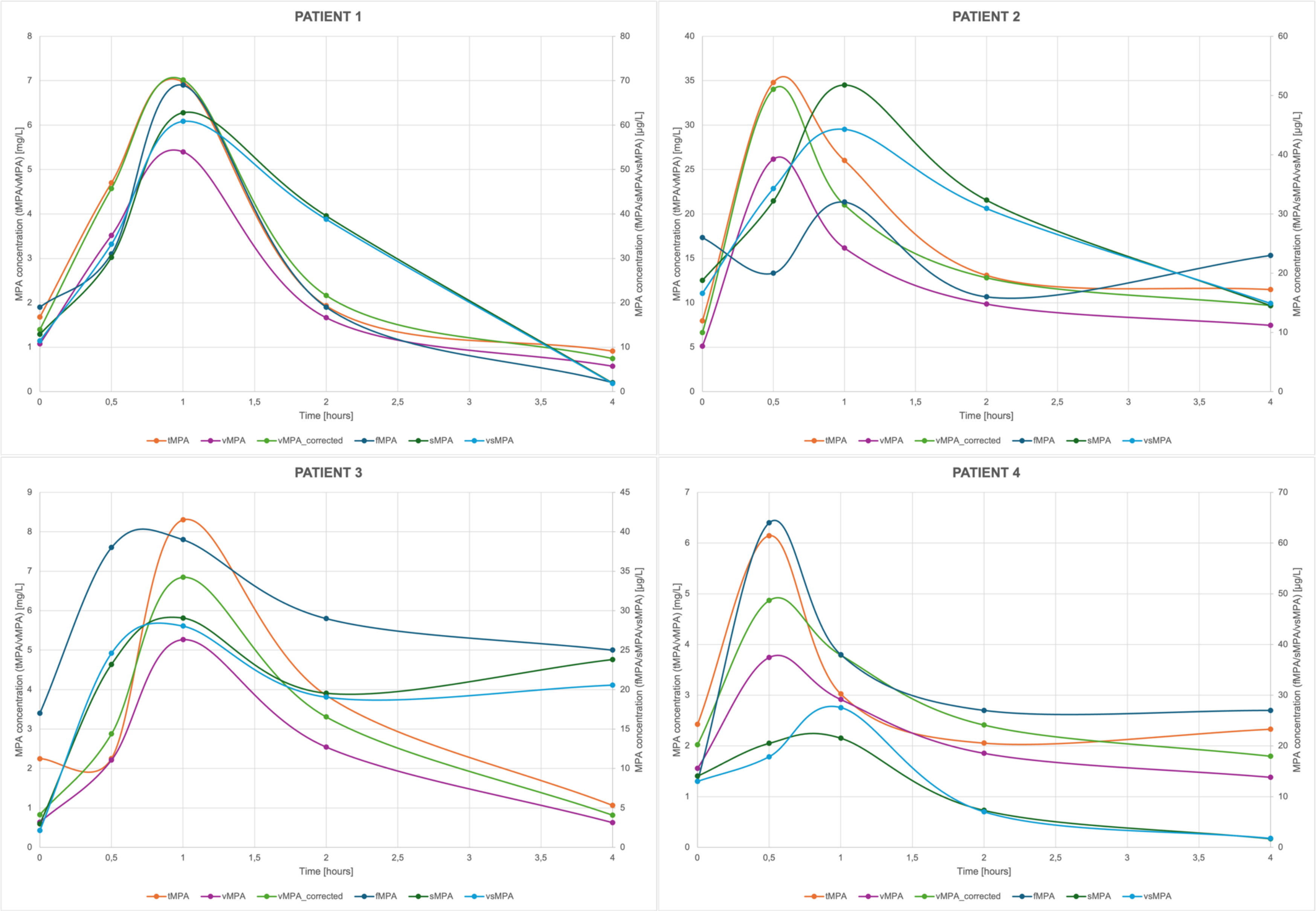
PATIENT 1
MPA concentration (tMPA/vMPA) [mg/L]
MPA concentration (fMPA/sMPA/vsMPA) [µg/L]
Time [hours]
tMPA
vMPA
vMPA_corrected
fMPA
sMPA
vsMPA
PATIENT 2
MPA concentration (tMPA/vMPA) [mg/L]
MPA concentration (fMPA/sMPA/vsMPA) [µg/L]
Time [hours]
tMPA
vMPA
vMPA_corrected
fMPA
sMPA
vsMPA
PATIENT 3
MPA concentration (tMPA/vMPA) [mg/L]
MPA concentration (fMPA/sMPA/vsMPA) [µg/L]
Time [hours]
tMPA
vMPA
vMPA_corrected
fMPA
sMPA
vsMPA
PATIENT 4
MPA concentration (tMPA/vMPA) [mg/L]
MPA concentration (fMPA/sMPA/vsMPA) [µg/L]
Time [hours]
tMPA
vMPA
vMPA_corrected
fMPA
sMPA
vsMPA

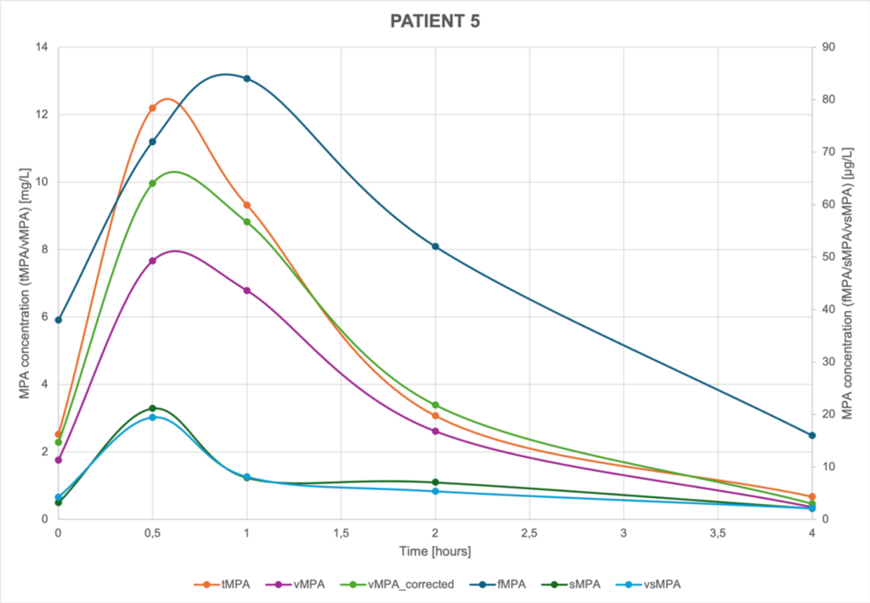
PATIENT 5
MPA concentration (tMPA/vMPA) [mg/L]
MPA concentration (fMPA/sMPA/vsMPA) [µg/L]
Time [hours]
tMPA
vMPA
vMPA_corrected
fMPA
sMPA
vsMPA

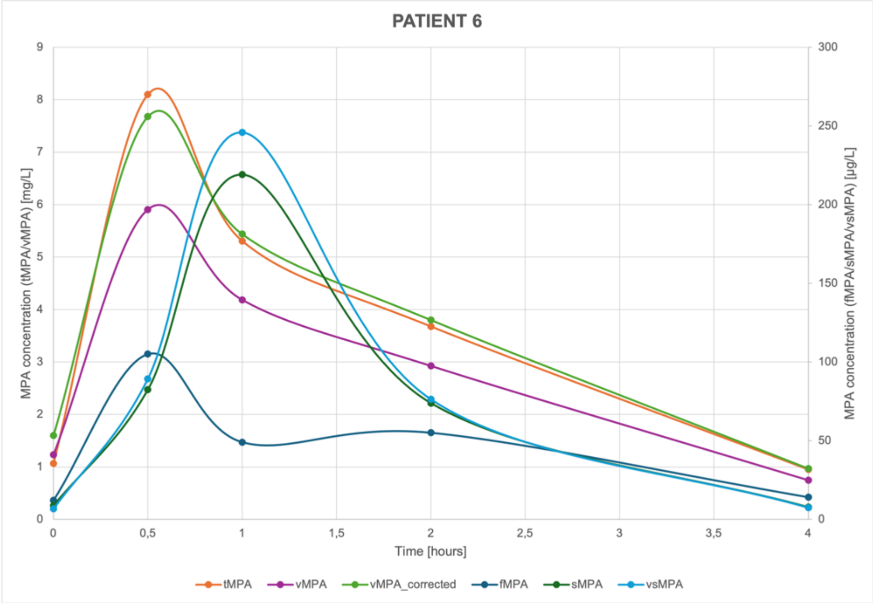
PATIENT 6
MPA concentration (tMPA/vMPA) [mg/L]
MPA concentration (fMPA/sMPA/vsMPA) [µg/L]
Time [hours]
tMPA
vMPA
vMPA_corrected
fMPA
sMPA
vsMPA

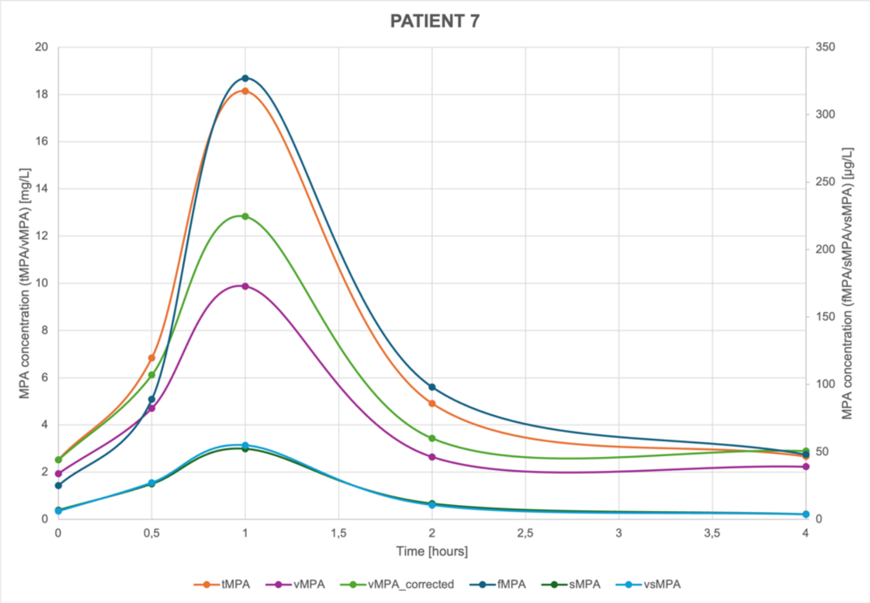
PATIENT 7
MPA concentration (tMPA/vMPA) [mg/L]
MPA concentration (fMPA/sMPA/vsMPA) [µg/L]
Time [hours]
tMPA
vMPA
vMPA_corrected
fMPA
sMPA
vsMPA

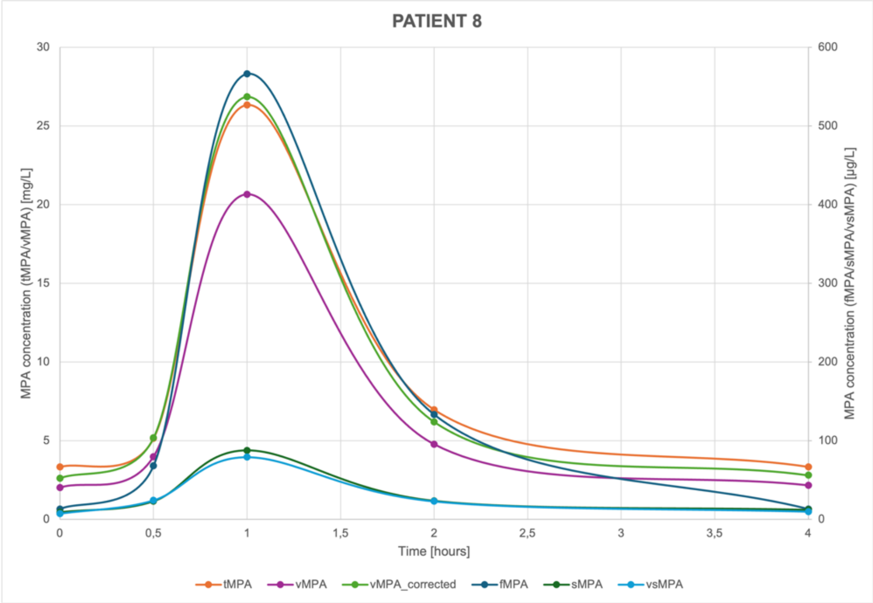
PATIENT 8
MPA concentration (tMPA/vMPA) [mg/L]
MPA concentration (fMPA/sMPA/vsMPA) [µg/L]
Time [hours]
tMPA
vMPA
vMPA_corrected
fMPA
sMPA
vsMPA

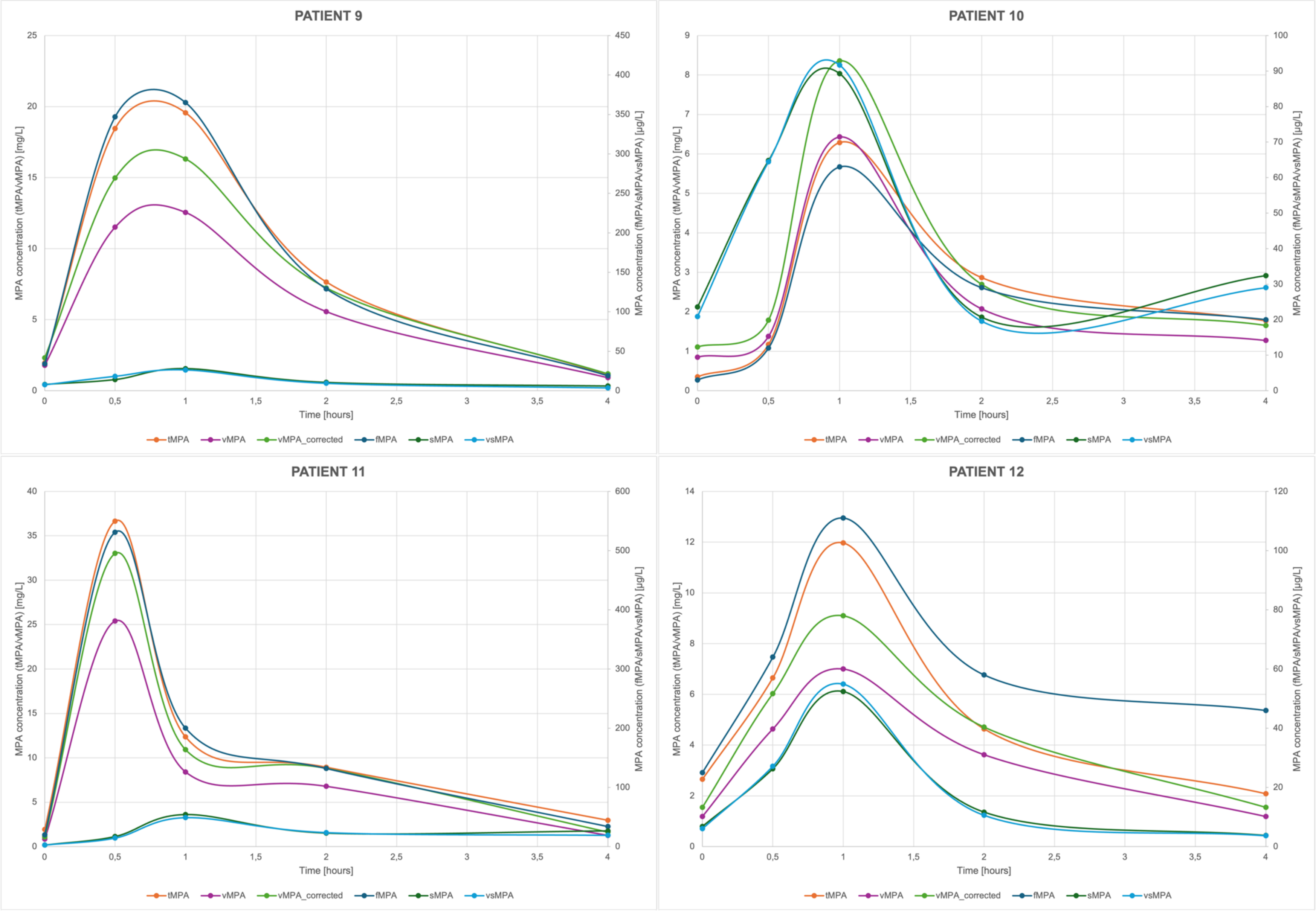

PATIENT 9
PATIENT 10
PATIENT 11
PATIENT 12
MPA concentration (tMPA/vMPA) [mg/L]
MPA concentration (fMPA/sMPA/vsMPA) [µg/L]
Time [hours]
tMPA
vMPA
vMPA_corrected
fMPA
sMPA
vsMPA

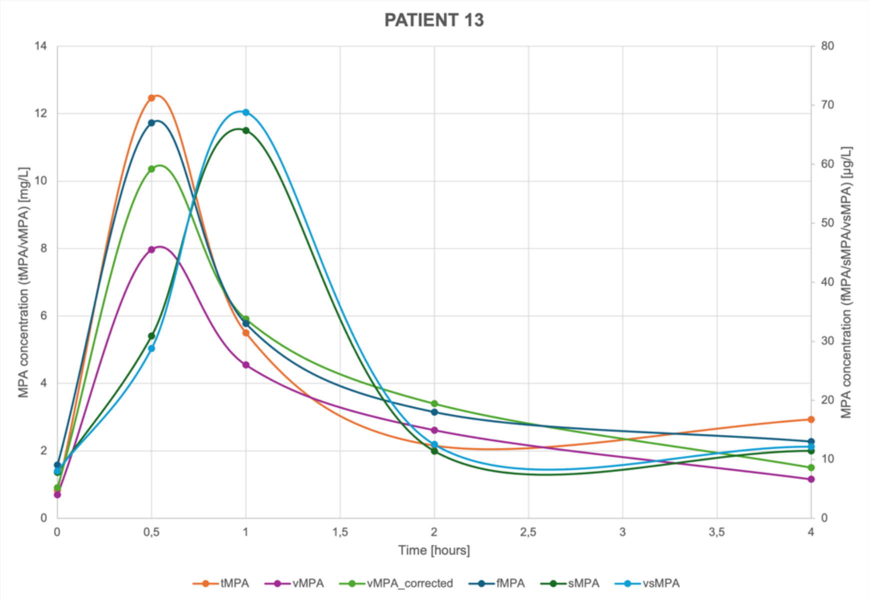
PATIENT 13
MPA concentration (tMPA/vMPA) [mg/L]
MPA concentration (fMPA/sMPA/vsMPA) [µg/L]
Time [hours]
tMPA
vMPA
vMPA_corrected
fMPA
sMPA
vsMPA

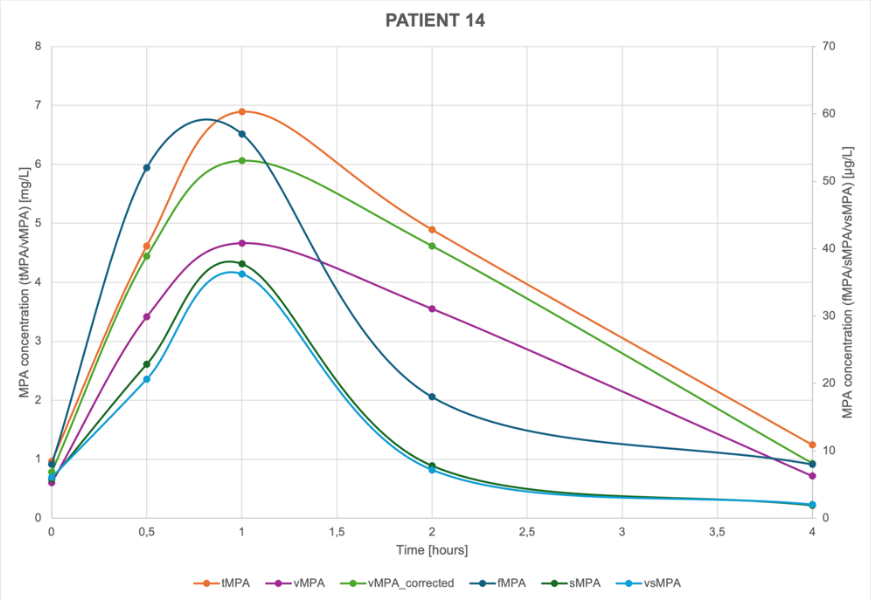
PATIENT 14
MPA concentration (tMPA/vMPA) [mg/L]
MPA concentration (fMPA/sMPA/vsMPA) [µg/L]
Time [hours]
tMPA
vMPA
vMPA_corrected
fMPA
sMPA
vsMPA

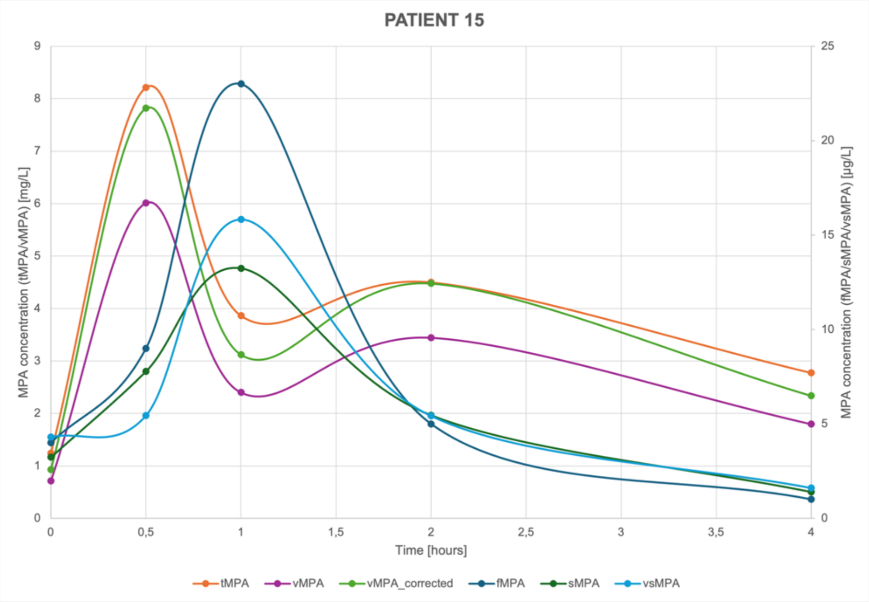
PATIENT 15
MPA concentration (tMPA/vMPA) [mg/L]
MPA concentration (fMPA/sMPA/vsMPA) [µg/L]
Time [hours]
tMPA
vMPA
vMPA_corrected
fMPA
sMPA
vsMPA

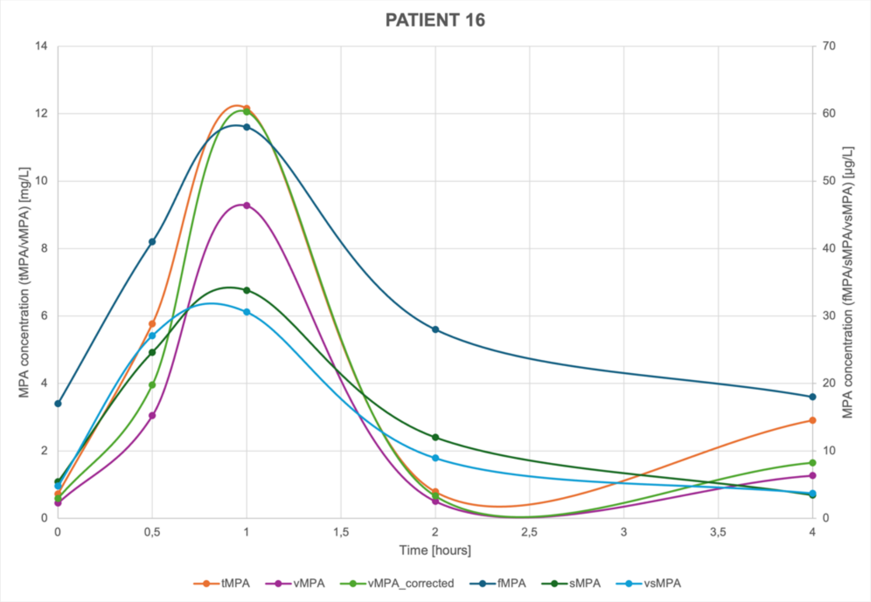
PATIENT 16
MPA concentration (tMPA/vMPA) [mg/L]
MPA concentration (fMPA/sMPA/vsMPA) [µg/L]
Time [hours]
tMPA
vMPA
vMPA_corrected
fMPA
sMPA
vsMPA

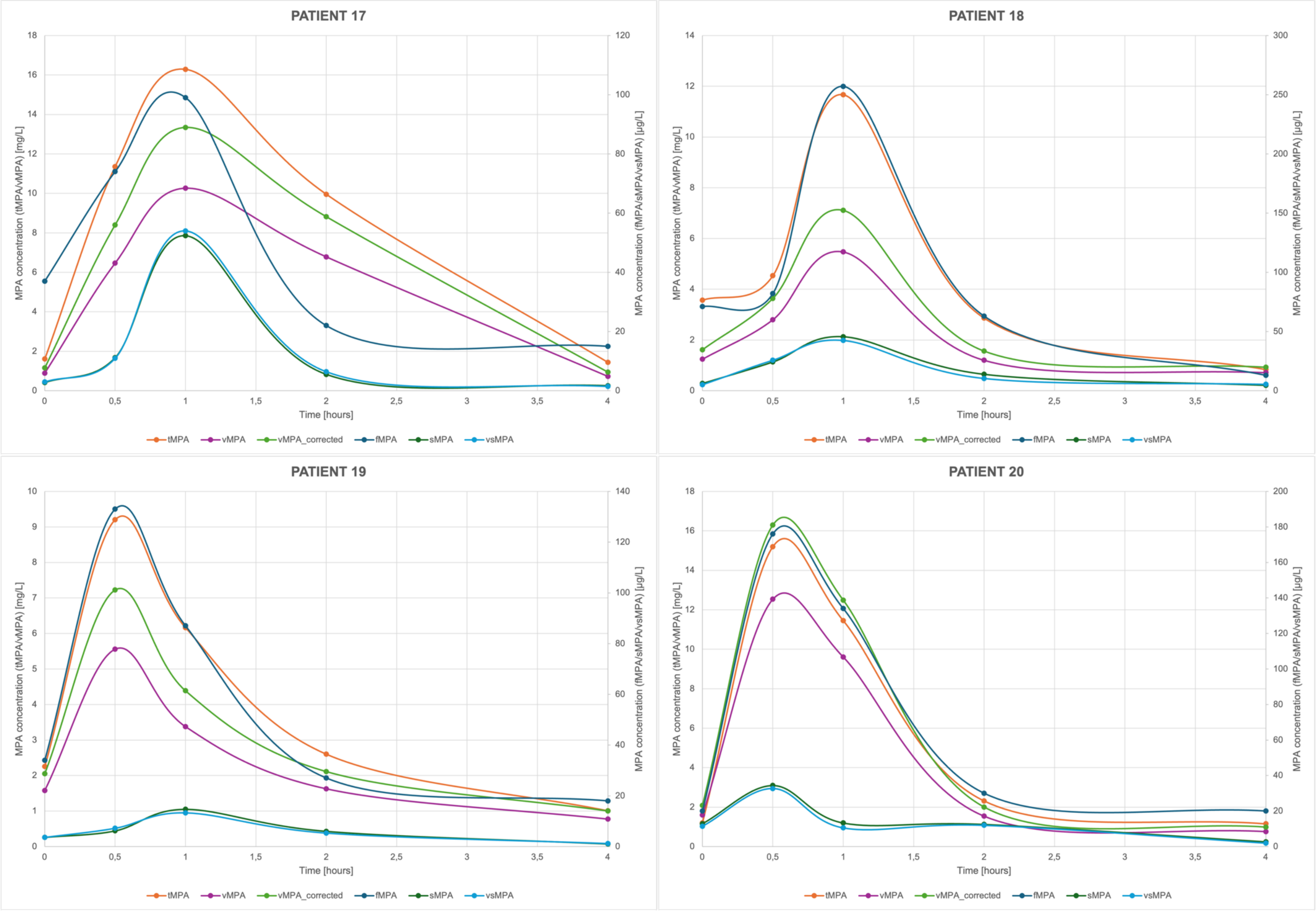


**Figure S4.** The individual PK profile plots of MPA levels in the tested matrices for each patient enrolled in the study (designations in the legend). The scale of the left axis refers to tMPA and vMPA concentrations, while the scale of the right axis refers to fMPA, sMPA, and vsMPA concentrations. tMPA – total MPA (mycophenolic acid) concentration in plasma, vMPA – MPA concentration in capillary blood collected using Mitra™-VAMS device, vMPA_corrected – recalculated MPA concentration (to estimated plasma level), fMPA – unbound MPA concentration, sMPA – wet salivary MPA concentration, vsMPA – dried salivary MPA concentration.

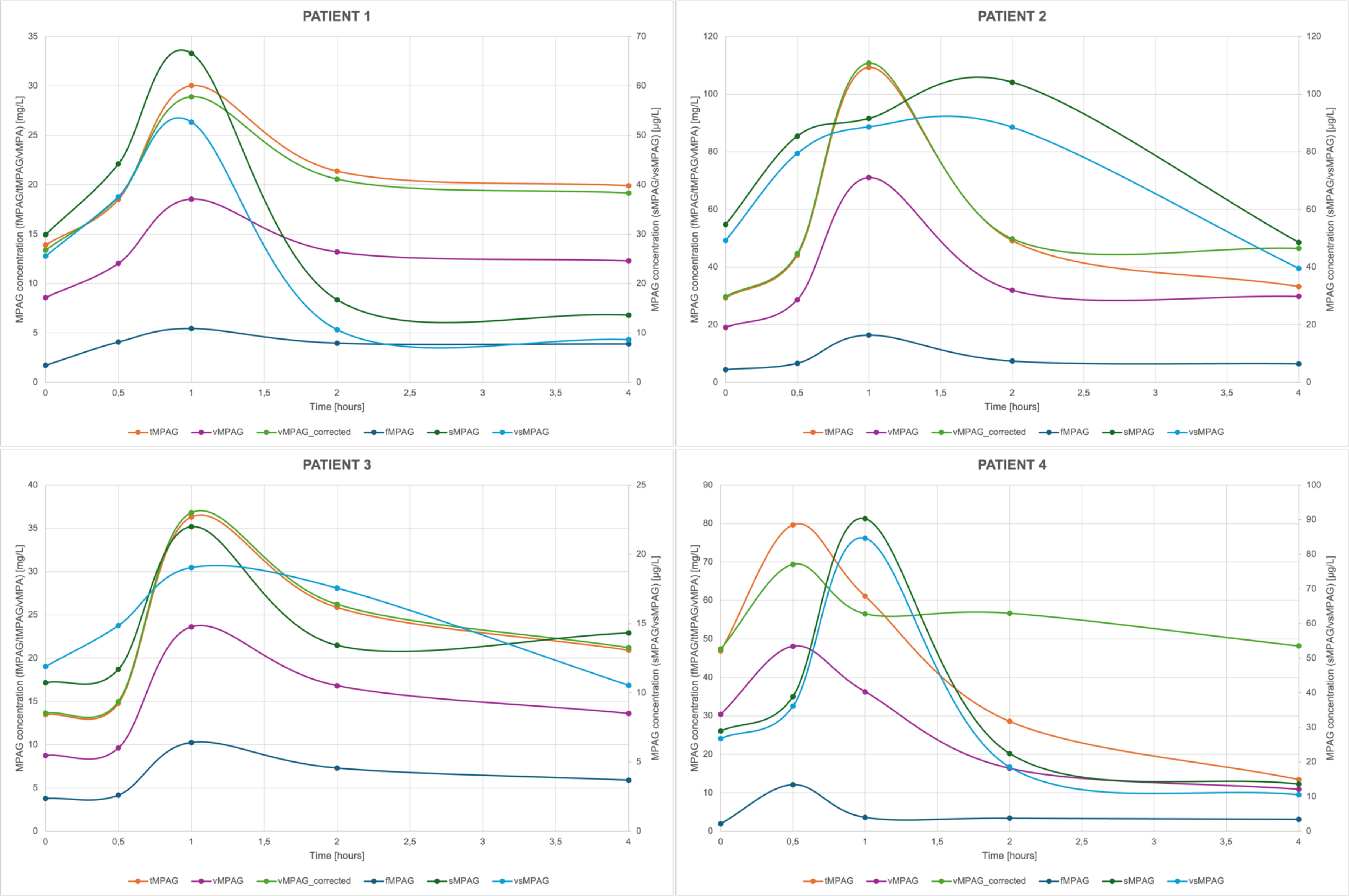
PATIENT 1
MPAG concentration (fMPAG/tMPAG/vMPA) [mg/L]
MPAG concentration (sMPAG/vsMPAG) [µg/L]
Time [hours]
tMPAG
vMPAG
vMPAG_corrected
fMPAG
sMPAG
vsMPAG
PATIENT 2
MPAG concentration (fMPAG/tMPAG/vMPA) [mg/L]
MPAG concentration (sMPAG/vsMPAG) [µg/L]
Time [hours]
tMPAG
vMPAG
vMPAG_corrected
fMPAG
sMPAG
vsMPAG
PATIENT 3
MPAG concentration (fMPAG/tMPAG/vMPA) [mg/L]
MPAG concentration (sMPAG/vsMPAG) [µg/L]
Time [hours]
tMPAG
vMPAG
vMPAG_corrected
fMPAG
sMPAG
vsMPAG
PATIENT 4
MPAG concentration (fMPAG/tMPAG/vMPA) [mg/L]
MPAG concentration (sMPAG/vsMPAG) [µg/L]
Time [hours]
tMPAG
vMPAG
vMPAG_corrected
fMPAG
sMPAG
vsMPAG

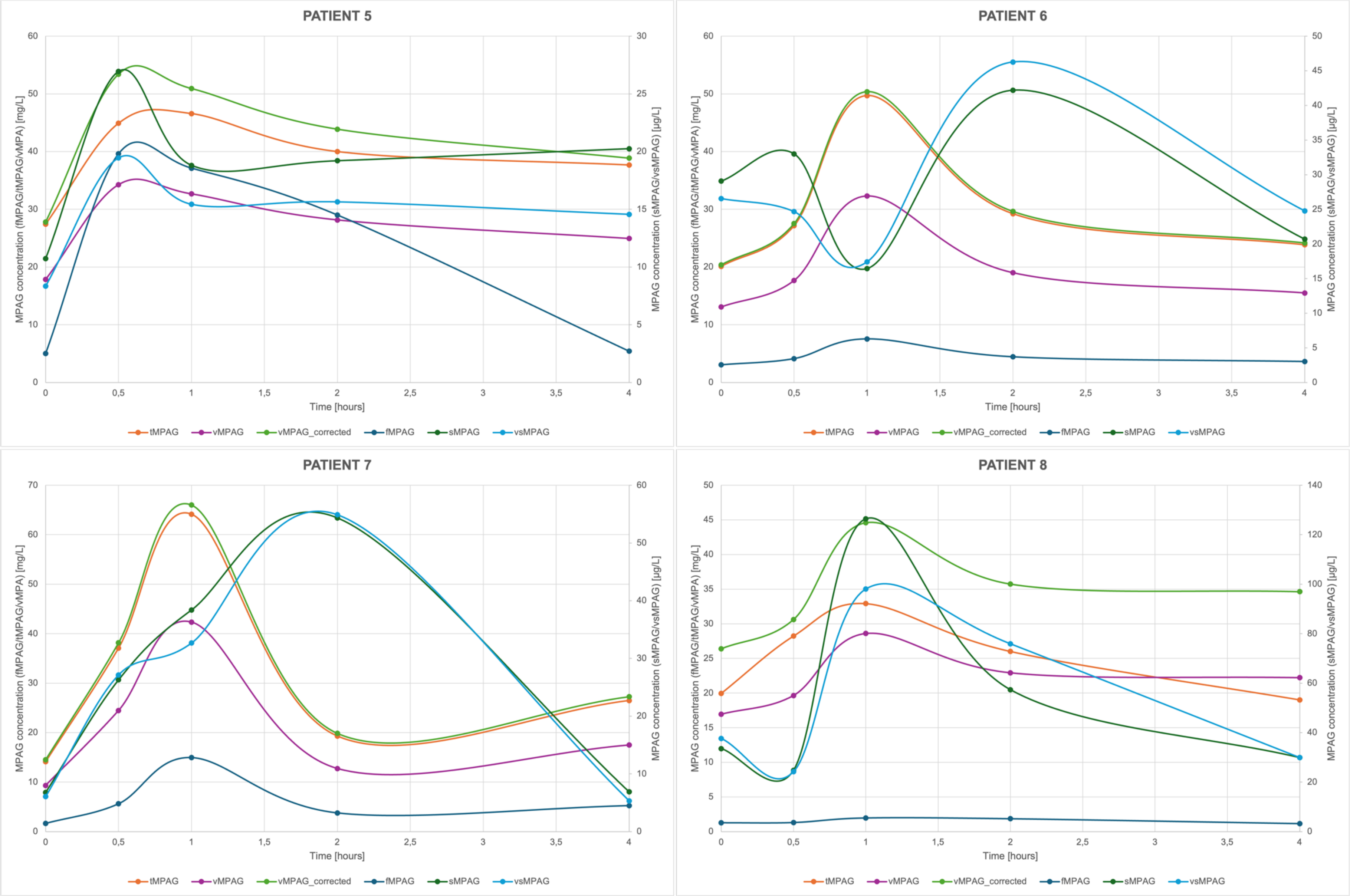
PATIENT 5
PATIENT 6
PATIENT 7
PATIENT 8
MPAG concentration (fMPAG/tMPAG/vMPAG/vMPA) [mg/L]
MPAG concentration (sMPAG/vsMPAG) [µg/L]
Time [hours]
tMPAG
vMPAG
vMPAG_corrected
fMPAG
sMPAG
vsMPAG

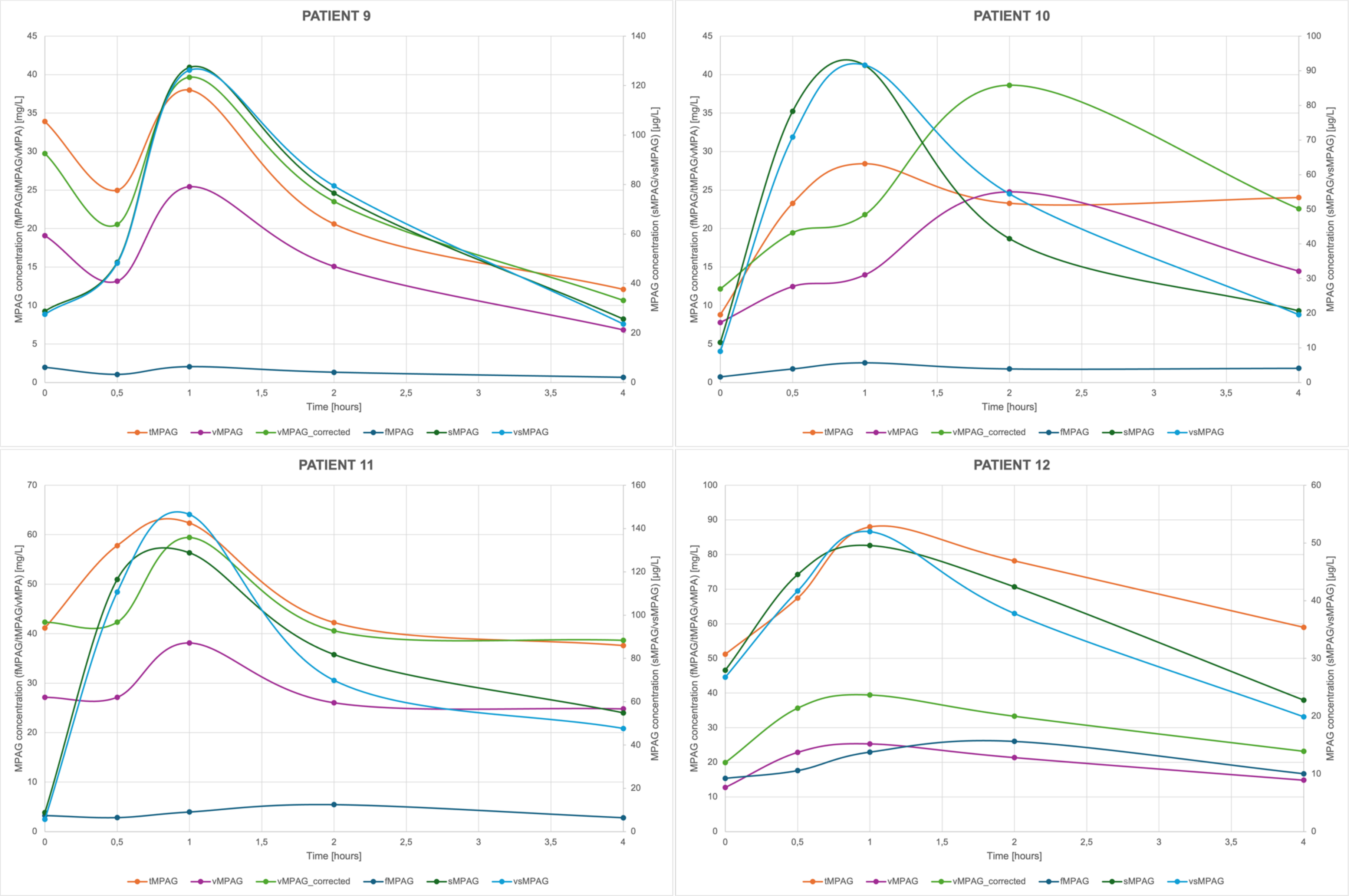
PATIENT 9
MPAG concentration (fMPAG/tMPAG/vMPA) [mg/L]
MPAG concentration (sMPAG/vsMPAG) [µg/L]
Time [hours]
tMPAG
vMPAG
vMPAG_corrected
fMPAG
sMPAG
vsMPAG
PATIENT 10
MPAG concentration (fMPAG/tMPAG/vMPA) [mg/L]
MPAG concentration (sMPAG/vsMPAG) [µg/L]
Time [hours]
tMPAG
vMPAG
vMPAG_corrected
fMPAG
sMPAG
vsMPAG
PATIENT 11
MPAG concentration (fMPAG/tMPAG/vMPA) [mg/L]
MPAG concentration (sMPAG/vsMPAG) [µg/L]
Time [hours]
tMPAG
vMPAG
vMPAG_corrected
fMPAG
sMPAG
vsMPAG
PATIENT 12
MPAG concentration (fMPAG/tMPAG/vMPA) [mg/L]
MPAG concentration (sMPAG/vsMPAG) [µg/L]
Time [hours]
tMPAG
vMPAG
vMPAG_corrected
fMPAG
sMPAG
vsMPAG

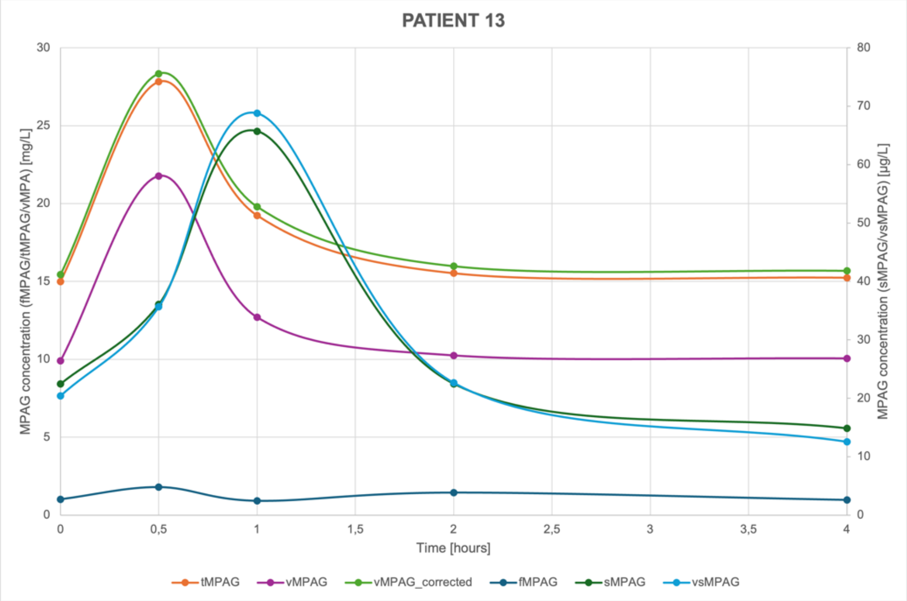
PATIENT 13
MPAG concentration (fMPAG/tMPAG/vMPA) [mg/L]
MPAG concentration (sMPAG/vsMPAG) [µg/L]
Time [hours]
0
0,5
1
1,5
2
2,5
3
3,5
4
tMPAG
vMPAG
vMPAG_corrected
fMPAG
sMPAG
vsMPAG

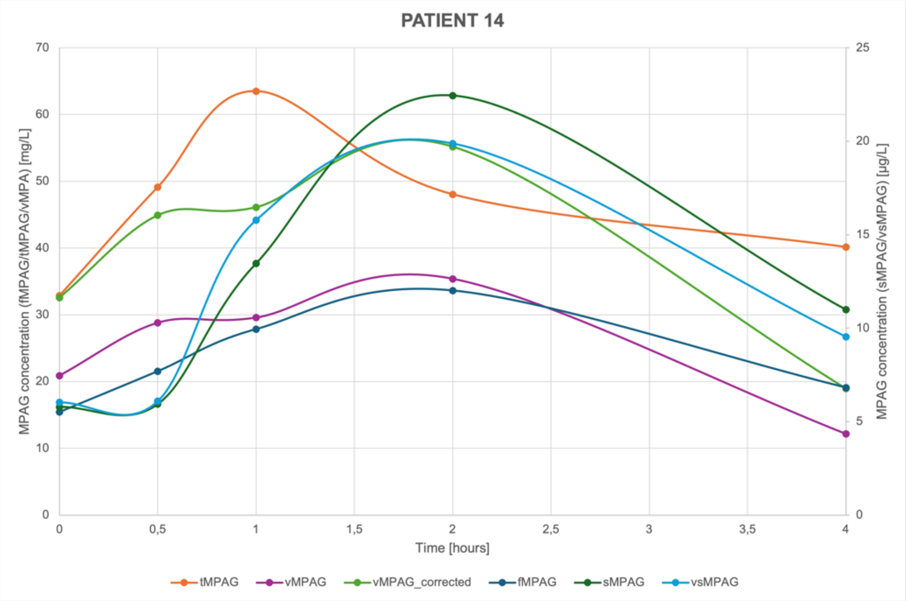
PATIENT 14
MPAG concentration (fMPAG/tMPAG/vMPA) [mg/L]
MPAG concentration (sMPAG/vsMPAG) [µg/L]
Time [hours]
0
0,5
1
1,5
2
2,5
3
3,5
4
tMPAG
vMPAG
vMPAG_corrected
fMPAG
sMPAG
vsMPAG

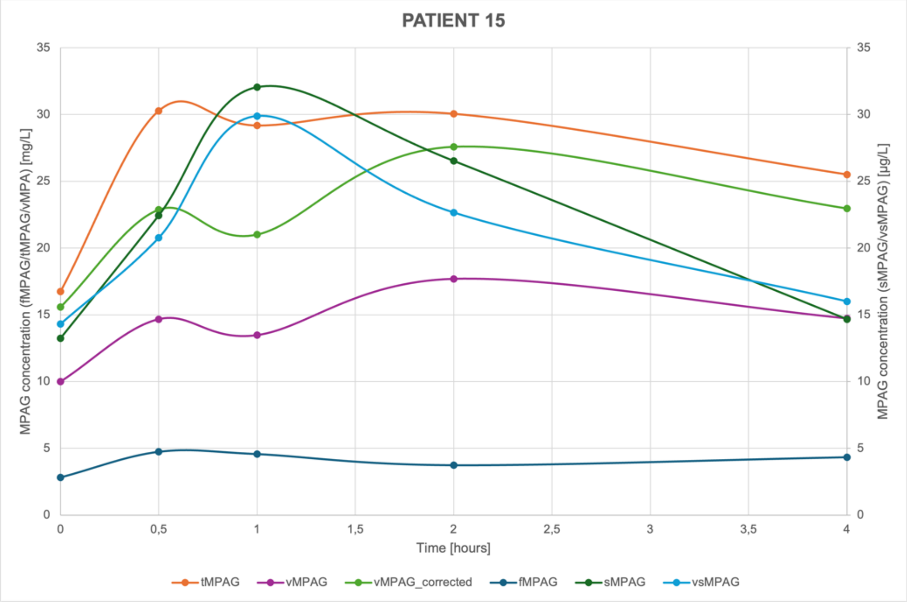
PATIENT 15
MPAG concentration (fMPAG/tMPAG/vMPA) [mg/L]
MPAG concentration (sMPAG/vsMPAG) [µg/L]
Time [hours]
0
0,5
1
1,5
2
2,5
3
3,5
4
tMPAG
vMPAG
vMPAG_corrected
fMPAG
sMPAG
vsMPAG

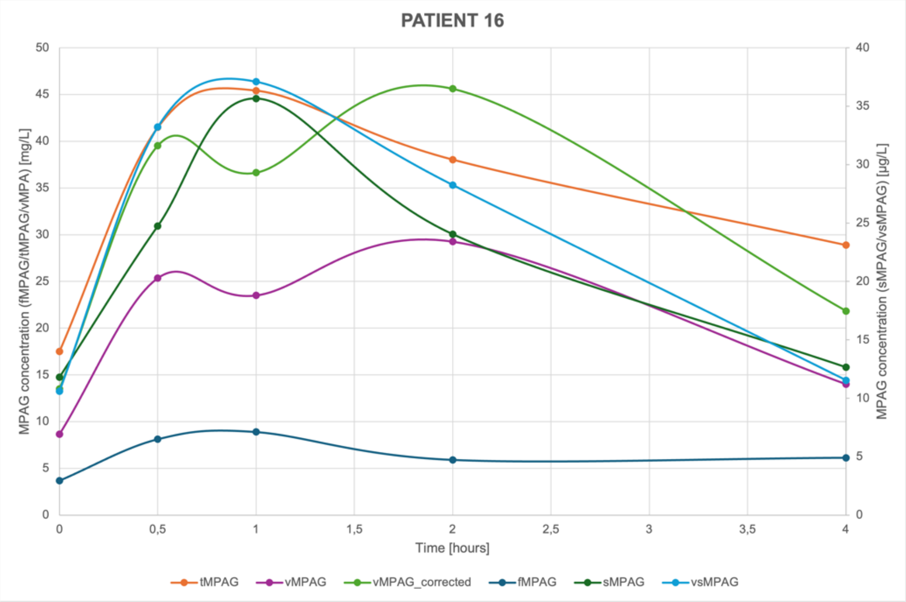
PATIENT 16
MPAG concentration (fMPAG/tMPAG/vMPA) [mg/L]
MPAG concentration (sMPAG/vsMPAG) [µg/L]
Time [hours]
0
0,5
1
1,5
2
2,5
3
3,5
4
tMPAG
vMPAG
vMPAG_corrected
fMPAG
sMPAG
vsMPAG

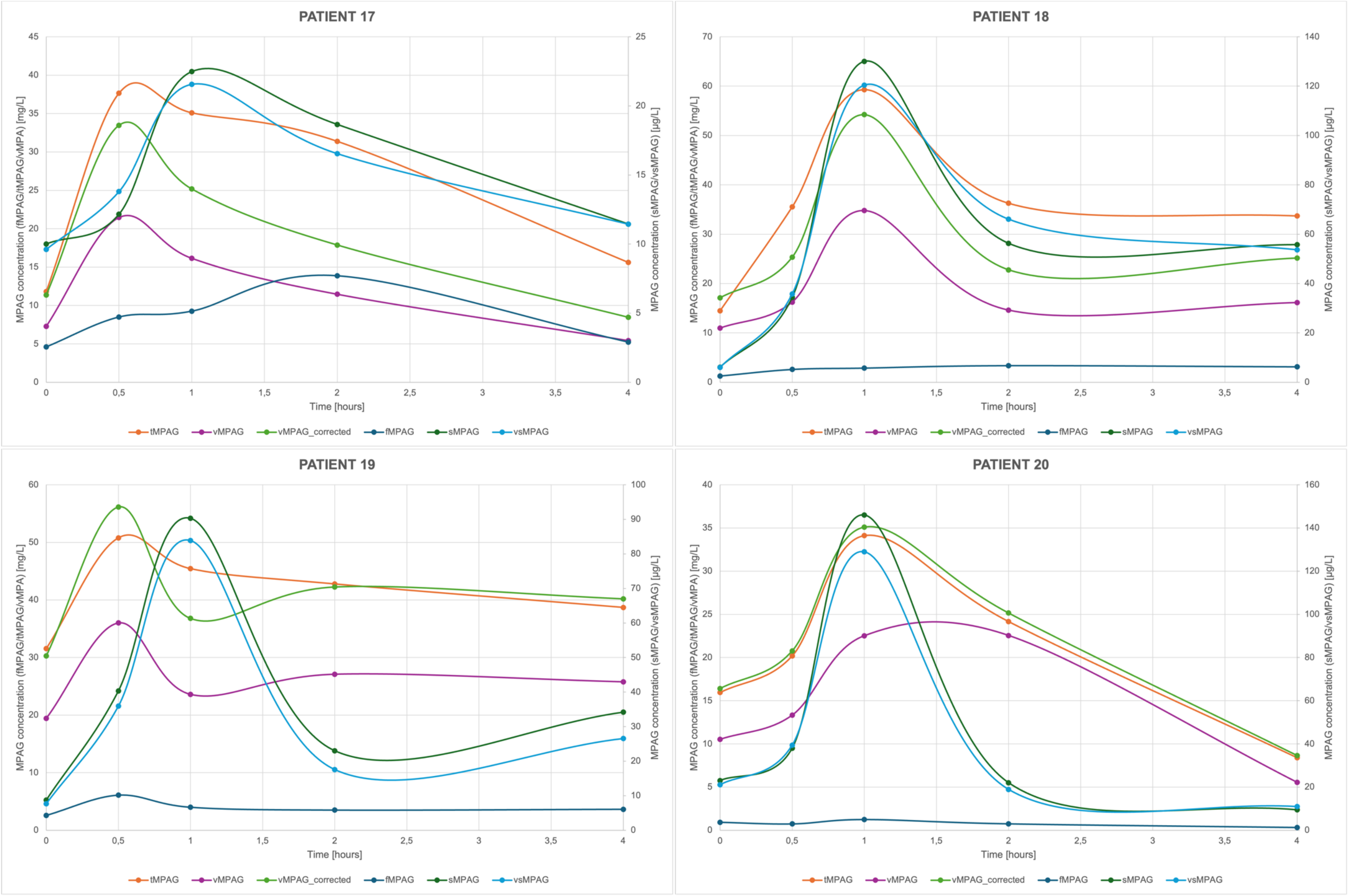


**Figure S5.** The individual PK profile plots of MPAG levels in the tested matrices for each patient enrolled in the study (designations in the legend). The scale of the left axis refers to fMPAG, tMPAG and vMPAG concentrations, while the scale of the right axis refers to sMPAG, and vsMPAG concentrations. tMPAG – total MPAG (mycophenolic acid glucuronide) concentration in plasma, vMPAG – MPAG concentration in capillary blood collected using Mitra™-VAMS device, vMPAG_corrected – recalculated MPAG concentration (to estimated plasma level), fMPAG – unbound MPAG concentration, sMPAG – wet salivary MPAG concentration, vsMPAG – dried salivary MPAG concentration.

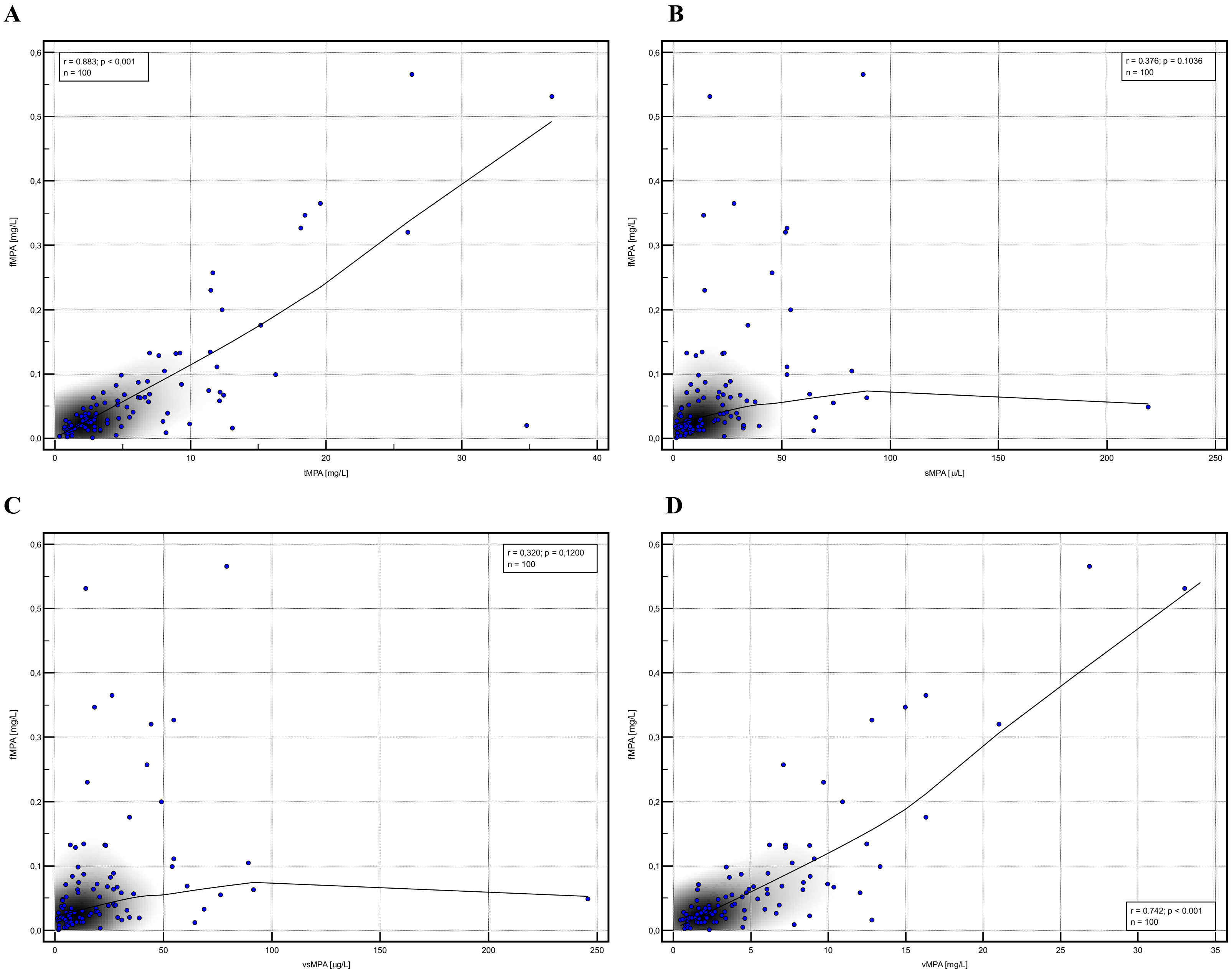
A
r = 0.883; p < 0,001
n = 100
fMPA [mg/L]
tMPA [mg/L]
B
r = 0.376; p = 0.1036
n = 100
fMPA [mg/L]
sMPA [µ/L]
C
r = 0,320; p = 0,1200
n = 100
fMPA [mg/L]
vsMPA [µg/L]
D
r = 0.742; p < 0.001
n = 100
fMPA [mg/L]
vMPA [mg/L]

**E**

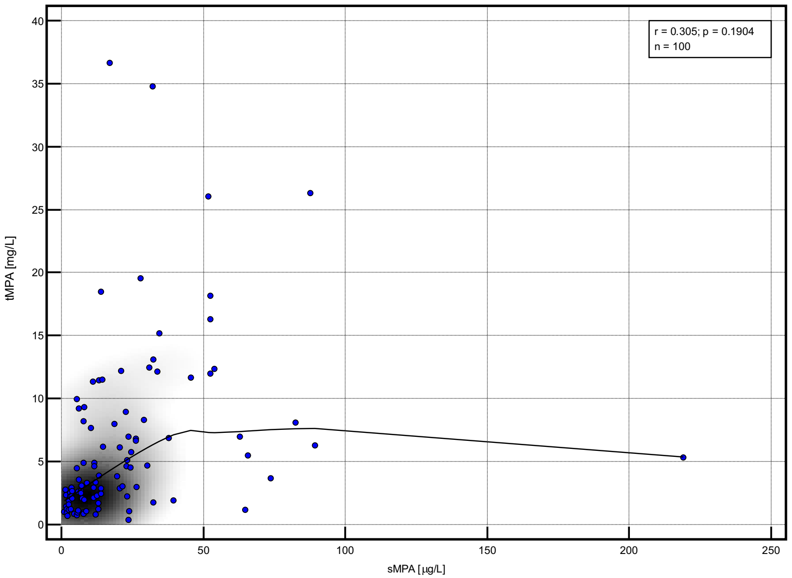
r = 0.305; p = 0.1904
n = 100
tMPA [mg/L]
sMPA [µg/L]

**F**

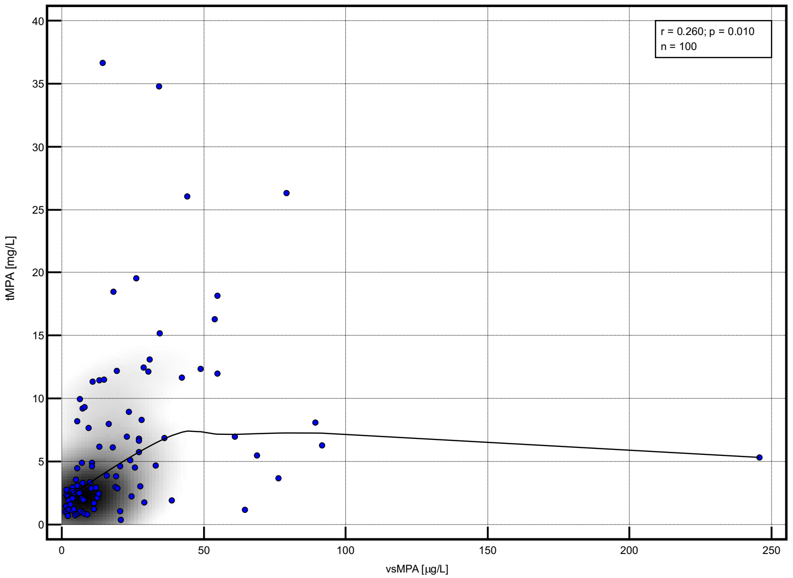
r = 0.260; p = 0.010
n = 100
tMPA [mg/L]
vsMPA [µg/L]

**G**

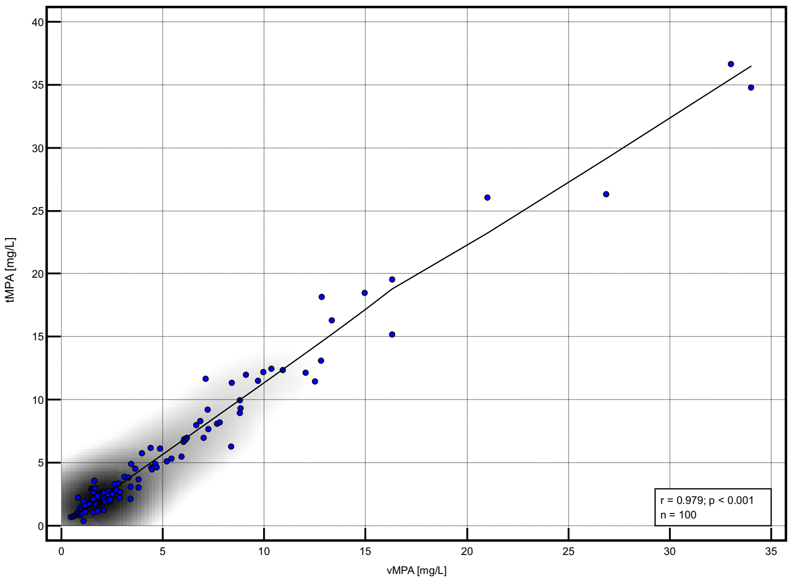
r = 0.979; p < 0.001
n = 100
tMPA [mg/L]
vMPA [mg/L]

**H**

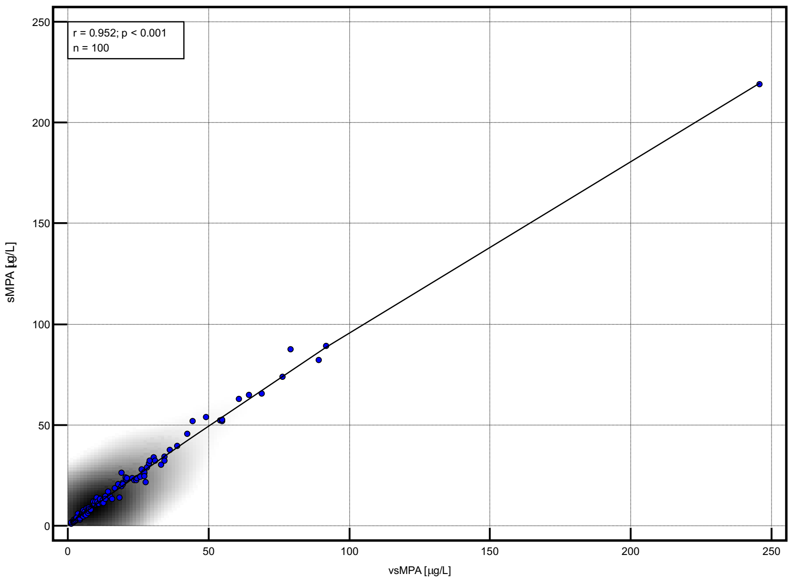
r = 0.952; p < 0.001
n = 100
sMPA [µg/L]
vsMPA [µg/L]

I

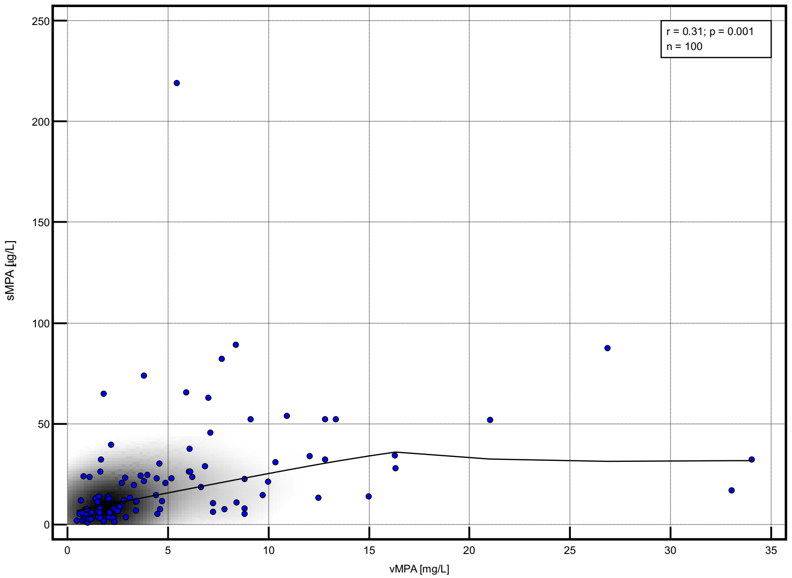
r = 0.31; p = 0.001
n = 100
sMPA [μg/L]
vMPA [mg/L]

J

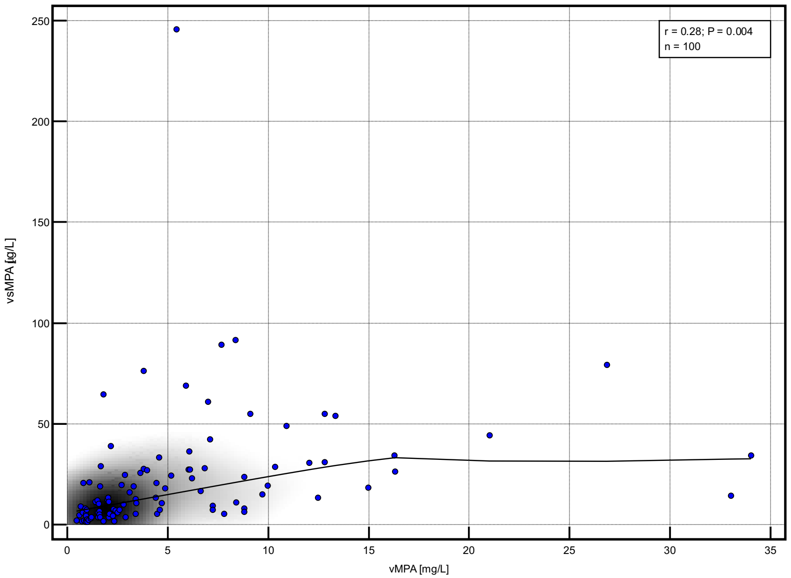
r = 0.28; P = 0.004
n = 100
vsMPA [μg/L]
vMPA [mg/L]

K

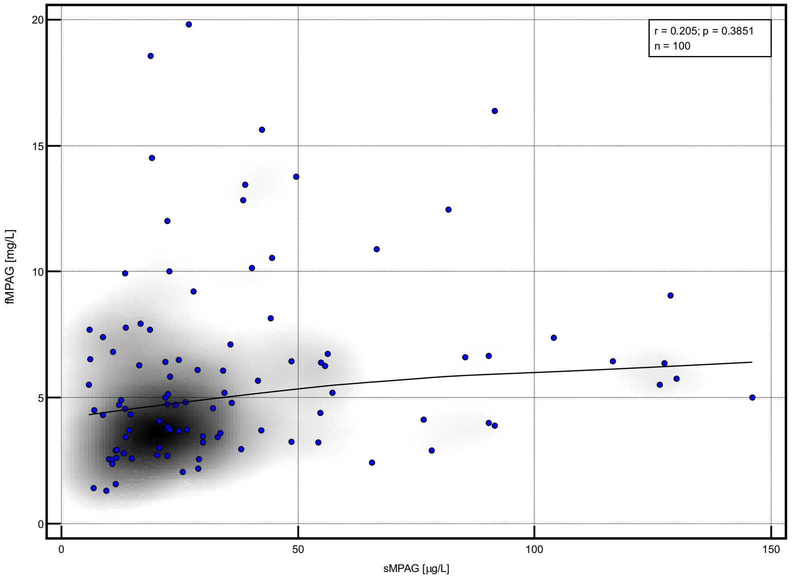
r = 0.205; p = 0.3851
n = 100
fMPAG [mg/L]
sMPAG [μg/L]

L

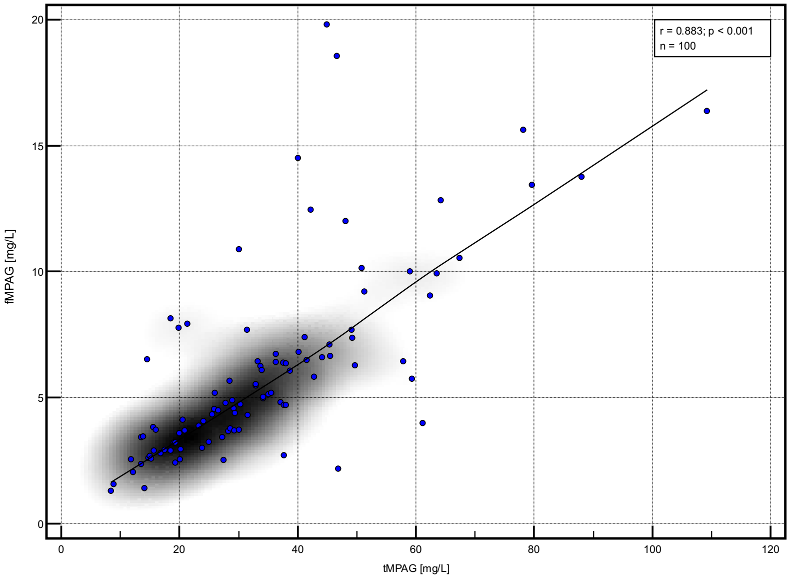
r = 0.883; p < 0.001
n = 100
fMPAG [mg/L]
tMPAG [mg/L]

**M**

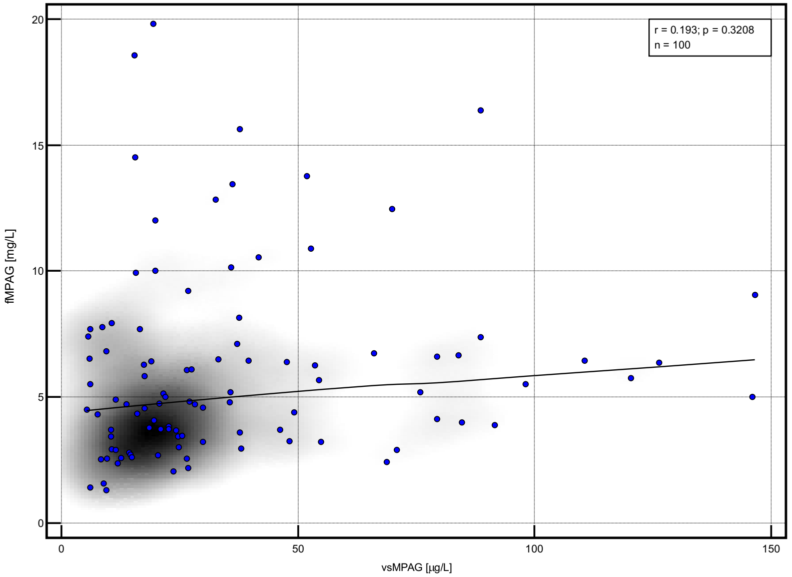
r = 0.193; p = 0.3208
n = 100
fMPAG [mg/L]
vsMPAG [µg/L]

**N**

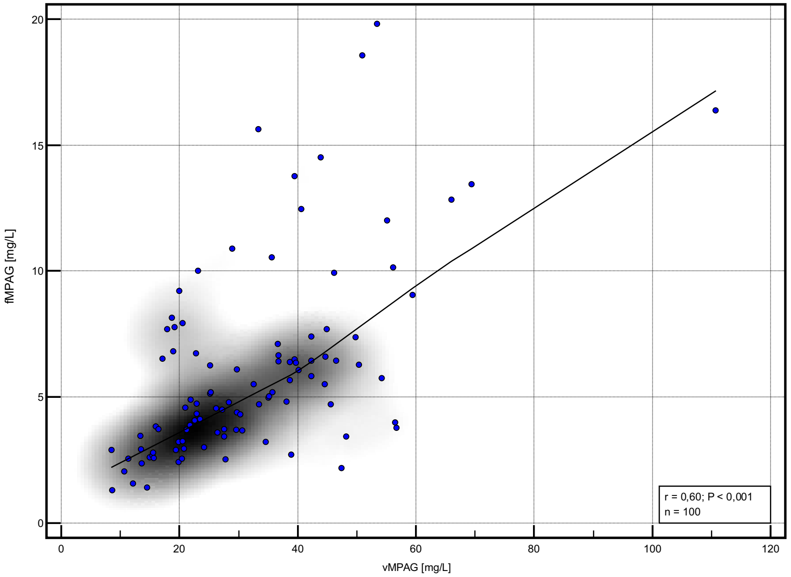
r = 0,60; P < 0,001
n = 100
fMPAG [mg/L]
vMPAG [mg/L]

**O**

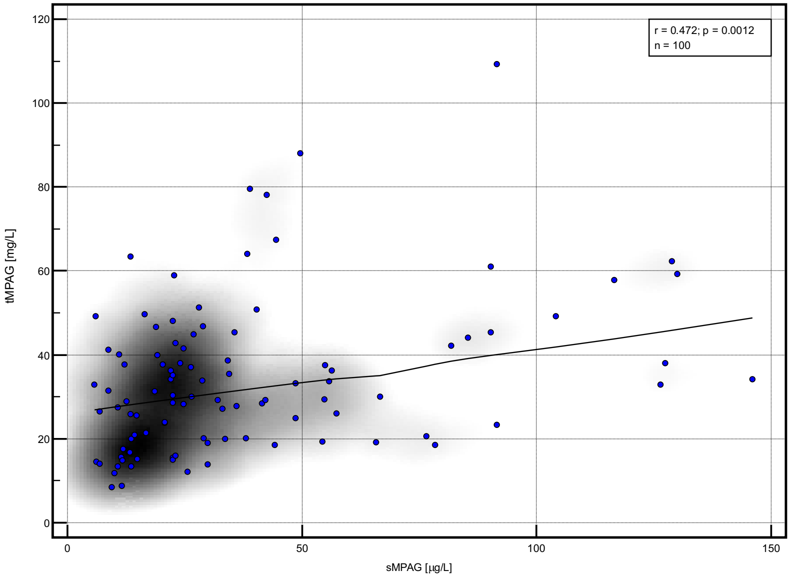
r = 0.472; p = 0.0012
n = 100
tMPAG [mg/L]
sMPAG [µg/L]

**P**

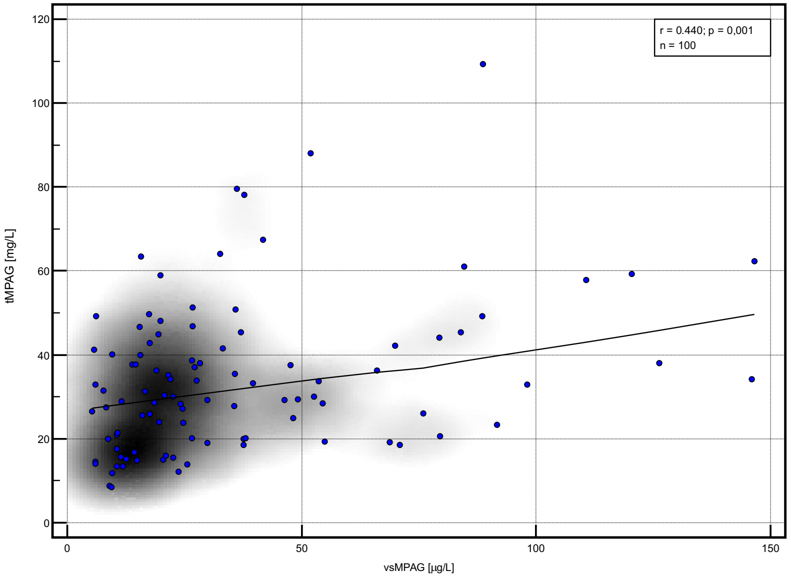
r = 0.440; p = 0,001
n = 100
tMPAG [mg/L]
vsMPAG [µg/L]

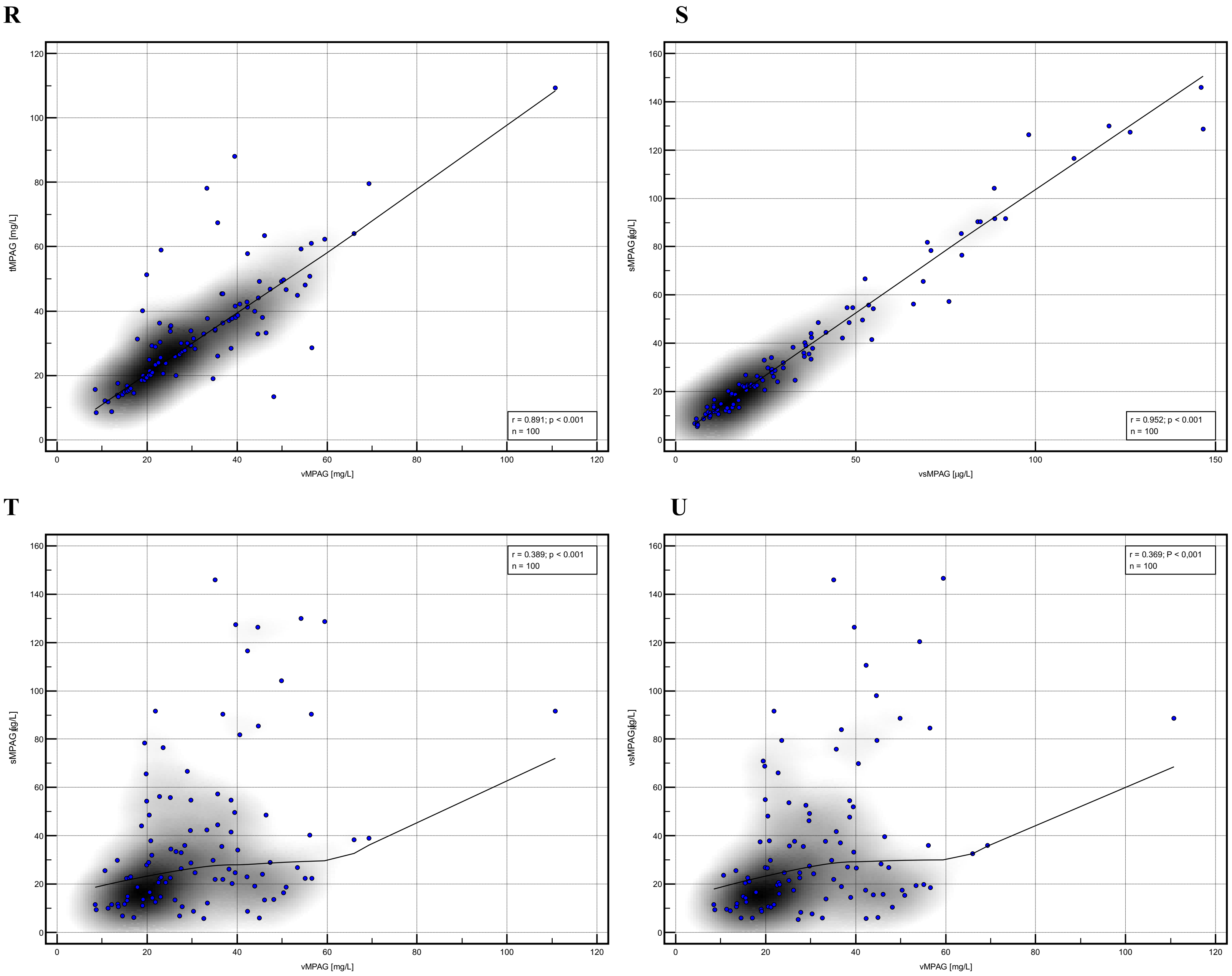


**Figure S6.** Correlation between MPA and MPAG levels in tested matrices (description of correlated data is presented as axis name with unit; designation of correlation coefficient with *p* value in the legend).
